# Managing healthcare employees' burnout through micro aspects of corporate social responsibility: A public health perspective

**DOI:** 10.3389/fpubh.2022.1050867

**Published:** 2023-01-09

**Authors:** Rongxin Chen, Wei Liu

**Affiliations:** ^1^School of Law, Xiamen University, Xiamen, China; ^2^Business School, Qingdao University, Qingdao, China

**Keywords:** burnout, healthcare, compassion, MCSR, employee behavior

## Abstract

**Background:**

Globally, an increasing number of healthcare workers (HCW) suffer from the issue of burnout (BO) annually. The critical issue of BO undermines the capacity of HCW to deliver superior healthcare services on the one end, it negatively affects the mental health of HCW on the other hand. Although HCW in developed and developing countries face the risk of BO, however, this issue is more critical in developing countries due to poor infrastructure, resources and social inequalities. The BO syndrome has recently been recognized as a public health concern, and new approaches are required to manage this epic, especially in healthcare management, effectively. In this respect, past research recognizes the role of corporate social responsibility (CSR) in influencing employee outcomes. Especially the micro aspects of CSR (MCSR) have recently received growing attention from academicians and practitioners. However, most existing MCSR investigations relate to the positive aspects of individual psychology, leaving the terrain unattended on how MCSR can help employees in reducing negative work outcomes for example, BO. To close this critical gap, the basic aim of this study is to investigate the relationship between MCSR and BO. Further, to understand the underlying mechanism of how and why MCSR may reduce employees' BO, this study introduces two mediators, work engagement (WE) and intrinsic motivation (IM) and one moderator, compassion at work (CW).

**Method:**

The data for the current study were gathered randomly from HCW serving in different hospitals of a developing country. Specifically, we collected the data in three separate waves. A self-administered questionnaire was used as a data collection instrument by following a paper-pencil methodology. The response rate in this study remained close to 64%. Both male and female HCW participated in this study. We validated the hypothesized relationships with the help of structural equation modeling in AMOS software.

**Results:**

The results confirmed that MCSR negatively predicts BO, and WE and IM mediated this relationship. Moreover, the moderating effect of CW was also confirmed.

**Conclusion:**

The findings of this study help healthcare administrators to mitigate the epic of BO among HCW by carefully planning and executing MCSR policies.

## 1. Introduction

Burnout (BO) has been a pressing issue in the literature on organizational management (1, 2). Although BO may exist in any profession, the prevalence of BO in healthcare workers (HCW) is high compared to other professions (3, 4). Characterized by different issues, including packed working routines, demanding job requirements, work overload, and irregular work timings, a healthcare environment puts employees at a higher risk of being burned-out (5). Conceptually, BO is assumed as a reaction of an individual in response to long-term work stress marked by depersonalization, reduced level of personal accomplishment and emotional exhaustion (6). Although the emergence of COVID-19 has accelerated the BO rate among HCW worldwide (7, 8), the National Academy of Medicine had already specified that BO in the healthcare system had reached a crisis level (9, 10). As one of the pressing issues in healthcare management, an increased level of BO not only puts the physical and mental health of employees at risk but also negatively affects the whole healthcare system of a nation, undermining the collective public health and wellbeing (11).

Perhaps Maslach Burnout Inventory (MBI) has been recognized as the leading measure of BO, which has been validated by the extensive research that has been conducted during the last 3 decades (12, 13). Even though fixing BO in an organization may require individual-level support for a burned-out person. Nevertheless, BO is mainly a distinctive occupational phenomenon (14), requiring a system-based (organizational level) solution for effective preparation for this black swan in healthcare. To spark this debate further, Jennifer (15) has argued that addressing BO by proposing a personal (individual) ban-aided solution may not be effective. Rather, a system-based approach can provide an effective and lasting solution. This system-based approach to fixing BO has started to flourish only recently, especially after the new definition of BO by WHO, in which it was clearly specified that BO is an occupational phenomenon and requires organizational interventions. Since the emergence of this new definition by WHO on BO, a plethora of behavioral scientists proposed different organizational level interventions to meager the effect of BO on employees' part. For example, the exploration of recent literature reveals that scholars have proposed different leadership models to reduce the risk of BO (16, 17). Similarly, recent evidence also suggests that organizational interventions for example, culture (18), human resource policies (19), and perceived organizational support (20) may help an organization in reducing employees' BO.

Relevant to the above discussion, we tend to focus on an under-researched area in the domain of employees' BO. To this end, the role of an organization's corporate social responsibility (CSR) engagement in influencing various employee outcomes has been highlighted several times by previous scholars (21–23). Generally, the concept of CSR relates to discretionary and context-specific interventions of an organization for the benefit of all stakeholders with respect to the triple bottom line effect, which includes economic, social and environmental responsibility and an organization (24). Although the research on the micro-aspect of CSR (MCSR) has flourished in the last decades, the outcomes of the MCSR-employee behavior relationship are very positive, indicating that MCSR can influence a range of employee behaviors (25, 26). Unquestionably MCSR is an organizational enabler that brings different benefits to an ethical organization. However, a critical gap still exists in the domain of the MCSR-employee behavior relationship. That is, to date, the bulk of previous CSR literature has focused on positive employee psychology by proposing MCSR as an enabler to foster positive employee attitudes and behaviors (27–29). However, it remains an udder-investigated terrain on how MCSR can help an organization in managing different negative employee outcomes. We do not intend to establish here that the literature on this stream is non-existent because some recent scholars have investigated MCSR to manage employee exhaustion (30), turnover intentions (31), stress (32) etc. Even discussion on BO exists in the MCSR framework (33, 34). Nevertheless, this sparse explanation is inadequate to reach a consensus. Moreover, the discussion on the underlying mechanism of how and why MCSR meager BO risk in an organization is not known, indicating another knowledge gap.

To spark the debate on employee BO, this study has two main objectives. First, this study aims to enrich the growing body of knowledge by investigating how MCSR relates to employees' BO. As indicated above, previous research mostly focused on positive employee psychology in an MCSR framework, creating a critical knowledge gap by leaving the terrain of MCSR-BO unattended. Second, and most importantly, this study aims to explain the underlying mechanism betwixt MCSR-BO relationship with the help of two psychological factors as mediators: work engagement (WE) and intrinsic motivation (IM). At the same time, this study proposes the conditional indirect effect of compassion at work (CW) betwixt the mediated relationships of MCSR and BO *via* WE and IM. The reason to propose these psychological factors as mediators and moderator lies with the argument raised in the seminal work by Glavas (35), who emphasized the importance of understanding employee psychology in an MCSR framework with the help of mediators and moderators. Indeed, Glavas believed that the manifestation of mediator(s) and moderator(s) might explain the underlying mechanism of how and why CSR influences a specific employee-related outcome. Other scholars have also shared the same viewpoint (36, 37).

All in all, this study sparks the existing body of knowledge in the following ways. Firstly, this study is one of the fewest studies which relate MCSR with negative employee outcomes, especially from the perspective of BO in the healthcare sector. This study is important because BO is a critical issue in healthcare management that not only affects the mental health of HCW, but is also a concern of public health because HCW with poor mental health is likely to undermine the quality of patient healthcare delivery, which ultimately undermine the overall public health in a region, state or even a country (38, 39). With an increasing rate of BO in a healthcare system, the mental health of HCW worsens, undermining the public's ability to access healthcare treatment, making it harder for a nation to deal with a public health emergency, increasing health disparities, and ultimately creating a public health crisis. Therefore, there is a dire need to meager the effect of employee BO in healthcare.

Secondly, previously the phenomenon of BO, especially in a healthcare context, was mainly examined in developed countries. We do not undermine previously published work in this domain. However, our argument here is that the phenomenon of employee BO presents a worse situation in developing economies. The principal reason for this severity lies in the poor social and infrastructural support because compared to developed countries, the healthcare resources in most developing countries are scarce (40), placing the difficulty bar at a higher level for employees and increasing the risk to be burned-out. Therefore, studies conducted in developed countries may not reflect the case of developing countries. This indicates that separate studies are required from the standpoint of developing countries for a deeper understanding. Thirdly, this study contributes to the existing body of knowledge by proposing a robust model to explain the MCSR-BO relationship. The reason why we consider the proposed framework of this study as a robust model lies in the argument that this study considers different psychological and personal factors in a unified framework to predict employee BO in an MCSR framework.

The rest of this study has been estranged into four major sections. For instance, the theoretical grounding and relevant literature belong to the coming section, followed by the methodology, where we provide information about the population, sample, and data collection process. The next segment deals with data analysis to test the hypothesized relationships. The discussion section is the last, where we discuss the results relevant to previous studies with different implications. The potential limitations of this study and the conclusion have also been incorporated in this section. Reporting of this study followed the Strengthening the Reporting of Observational Studies in Epidemiology statement (41).

## 2. Theory and hypotheses

This study traces its theoretical roots in the theory of conservation of resources (TCOR) to explain the possible links between different variables in this study, especially between CSR and BO. This is an overarching theory in the literature on human resources and organizational management (42, 43). Particularly, BO literature has largely used this theory to explain the phenomenon of employee BO in different contexts (44, 45). Having its roots in the seminal work of Hobfoll (46), this theory proposes that a person is expected to gain, build and protect all kinds of resources (physical, psychological, and others) that he or she feels can be valuable to combat difficult or uncertain situations. Reflecting on the crux of this theory in a CSR framework, Lin and Liu (47) believed that BO manifests in an organization when employees lose valued resources, depletion of resources or a lack of gained resources. According to Hobfoll (48), valued resources may include acknowledgment of employees when they accomplish something, life meaning or purpose, a supportive environment, and a working place with a value-congruence betwixt employees and the organization. When employees feel that any of such resources are at stake, this perception of resource loss or depletion may trigger negative emotions among employees, increasing the likelihood for them to be burned-out. In this respect, an ethical organization, under its CSR philosophy, works to improve the mental health and wellbeing of all employees without any prejudice. Hence, employees working in an ethical organization are less likely to face an overwhelming burnout situation. The contextual resources provided by an ethical organization to the employees, provide them with added energy and strength which reduces the chance that the employees will be mere victim of BO while fixing a difficult situation in a workplace. Past researchers have also acknowledged the role of conservation of resources to determine BO, IM and WE of employees (49–51). We feel this is justifiable as BO, IM and WE all relate to the emotional component of human brain which rests on psychological resources for example vigor, agility and emotional robustness (48, 52). For all of such psychological resources, the MCSR philosophy of an organization is of profound importance because an ethical organization shows a genuine concern for the betterment of employees and takes every measure which can improve the mental health and wellbeing of employees. Conceptually, employees with improved mental health and wellbeing levels are expected to be less prone to the risk of BO. Therefore:

H1: MCSR policies of an ethical organization are expected to reduce the BO level among employees.

Literature suggests that the critical contributor to employees' BO is the persistent imbalance between the resources and the tasks required in a job (53). Employees are expected to be overwhelmed when they feel that the resources provided by their organization to fulfill a job task, for example, equipment, working environment, time, information, and training, are insufficient (54). Unfortunately, the current dynamic business environment, characterized by several workplace challenges, forces employees to bear the psychological brunt of improving organizational productivity, which ultimately increases the risk of BO among employees (55). What companies in the current age need to realize is the fact that the downward spiral effect on employees' health which is accompanied by BO, is not in any interest of an organization. Further, BO not only leads an organization toward a reduced level of productivity but, at the same time, it reduces the morale of employees, which may urge them to quit their job, ultimately making a dent in the overall performance of an organization (56). Empirical evidence proposes that employees with an improved level of engagement face less risk of being burned out in an organization (57, 58). There is a strong consensus among scholars that an engaged workforce is an ultimate key to organizational success (59, 60). A recent survey by GALLUP, which included more than 2,000 employees from different organizations in Japan, revealed that engaged employees show extra commitment and energy to perform their job and hence they are more resilient compared to disengaged workers. Therefore, such employees face less stress even during the difficult situation in a workplace (61). Glavas (35) also noted that employees with better WE bring their whole-self to a job and hence they show an increased level of energy, commitment and zest to complete their organizational assignment.

Interestingly there is increasing evidence that the CSR focus of an organization on the betterment of all stakeholders has a sparking effect on the engagement level of employees (62, 63). Especially scholars have well argued in favor of MCSR activities by an organization to spark their level of engagement (64, 65). Specifically, it was realized that different steps taken by an ethical organization for the wellness of employees are well respected by the employees and improve their mental health and wellbeing (66). Not only are the employees' internal CSR initiatives well taken by the employees, but the general CSR engagement of an organization for the external community and stakeholders also leaves a pleasant impression on employees (67). Employees feel pride in being the workers of an ethical organization, improving their engagement level, which then works as a foundation of added resources to combat BO. Hence it is theorized that:

H2: MCSR improves the level of WE among the employees.
H3: WE mediates between MCSR and BO of employees in an organization.

IM has been defined as a characteristic of a person in which he or she is internally motivated to achieve something for his or her inner satisfaction, not for external benefits (68). In this respect, it has been identified at many levels in the existing literature that individuals with a higher level of IM concentrate more on completing their tasks (69, 70). Additionally, IM assumed as one of the valued personal resources that resist different stressors, including BO (71, 72). Further, increasing evidence suggests that employees with an improved level of IM have an increasing level of personal resources and are expected to be more protected against the harmful effects of BO (73, 74).

Past evidence strongly supports that CSR can significantly spark the level of IM among employees (75, 76). Especially the existing literature well relates IM to MCSR activities of an organization (77). The ethical concern of a socially responsible organization to the welfare of employees urges them to respond positively to their organization. Therefore, employees show an extra level of energy and vigor to complete their job tasks. Hao et al. (76) reflect this by stating that the ethical engagement of an organization infuses a feeling of respect and trust among employees, which in turn influences IM positively. Moral norms and an ethical organization provide the employees with a reasonable justification to show an extra level of motivation in completing different tasks for an ethical organization (78). The mediating effect of IM in a CSR framework also preexists in the literature (79, 80). To conclude, as IM can negatively predict BO among employees (as a personal resource) and in an organizational milieu, IM is affected by the CSR engagement of an organization, we expect:

H4: IM of employees negatively predicts BO.
H5: IM mediates between MCSR and BO.

CW can be described as the willingness of an individual to devote time and effort to the wellbeing of others (81). CW is assumed to be the foundation of the healthcare profession (82). Indeed CW among HCW relates to the concern, suffering, and distress of patients and their families by taking different actions for their relief from a disease or illness (83). In a healthcare environment, CW is all about listening, respecting, and empathizing with the pain and suffering of patients. The literature argues that compassion and kindness are not only good for the receivers but are equally important for the providers and are the source of motivation for individuals to join a healthcare profession (84). When compassion exists in a healthcare organization, HCW are more effective with high morale and show greater motivation for patient care and safety (85). Compassionate individuals are more likely to show a greater congruence with organizational mission and values. The recent pandemic is a great example why CW is a core in medical profession. With the outbreak of pandemic, it was evident that a greater number of retired HCW joined their workplaces again to serve humanity. This altruistic attitude was largely initiated by their higher level of compassion. When employees hold a higher level of CW, they are more energetic and feel less resource constraints and hence are less likely to be burned-out soon in difficult situations (82).

CW helps employees in developing an environment in which they prefer the collective benefit of others. Interestingly the same collective benefit for others is the prime subject of CSR (63). Therefore, CW and CSR share the same concern, which is the collective benefit of others. The manifestation of CSR in an organization provides a context for the workers to build a higher level of CW on the employees' part. Hur et al. (79) mentioned that in response to the ethical engagement of an organization, employees respond positively by changing their extra-role behaviors, including pro-social behaviors. Nazir and Islam (63) believed that an ethical organization's social engagement could determine employees' CW.

Past evidence suggests that compassionate employees are better engaged in their work because their enhanced level of CW creates a positive emotional state among employees (86). Moreover, compassionate employees look for the collective benefit of others rather than promoting their own interest in an organization because the process of compassion involves sympathy for others' suffering (87). Consequently, CW provides a buffering effect for enhancing the WE of employees in an ethical organization. Previous literature also indicates that compassionate people show a higher level of engagement with their job (88, 89).

IM and CW have special meanings in a healthcare context because IM on one end, motivates HCW to serve humanity for their inner satisfaction (inner reward), CW on the other hand, urges employees to feel the suffering of others and helps them to get rid of such sufferings (90). Hence it can be thought that compassionate people show more IM to help other for their inner satisfaction. Indeed CW is other-oriented approach because it urges an individual to pay attention to the sufferings of others. In this regard, CSR is also other-oriented approach, therefore, we expect that in an ethical organization, CW can produce a buffering effect between the relationship of MCSR and IM. Thus we propose the following hypotheses:

H6: CW moderates the mediated relationship between MCR and BO *via* WE.
H7: CW moderates the mediated relationship between MCR and BO *via* IM.

**Figure 1:**
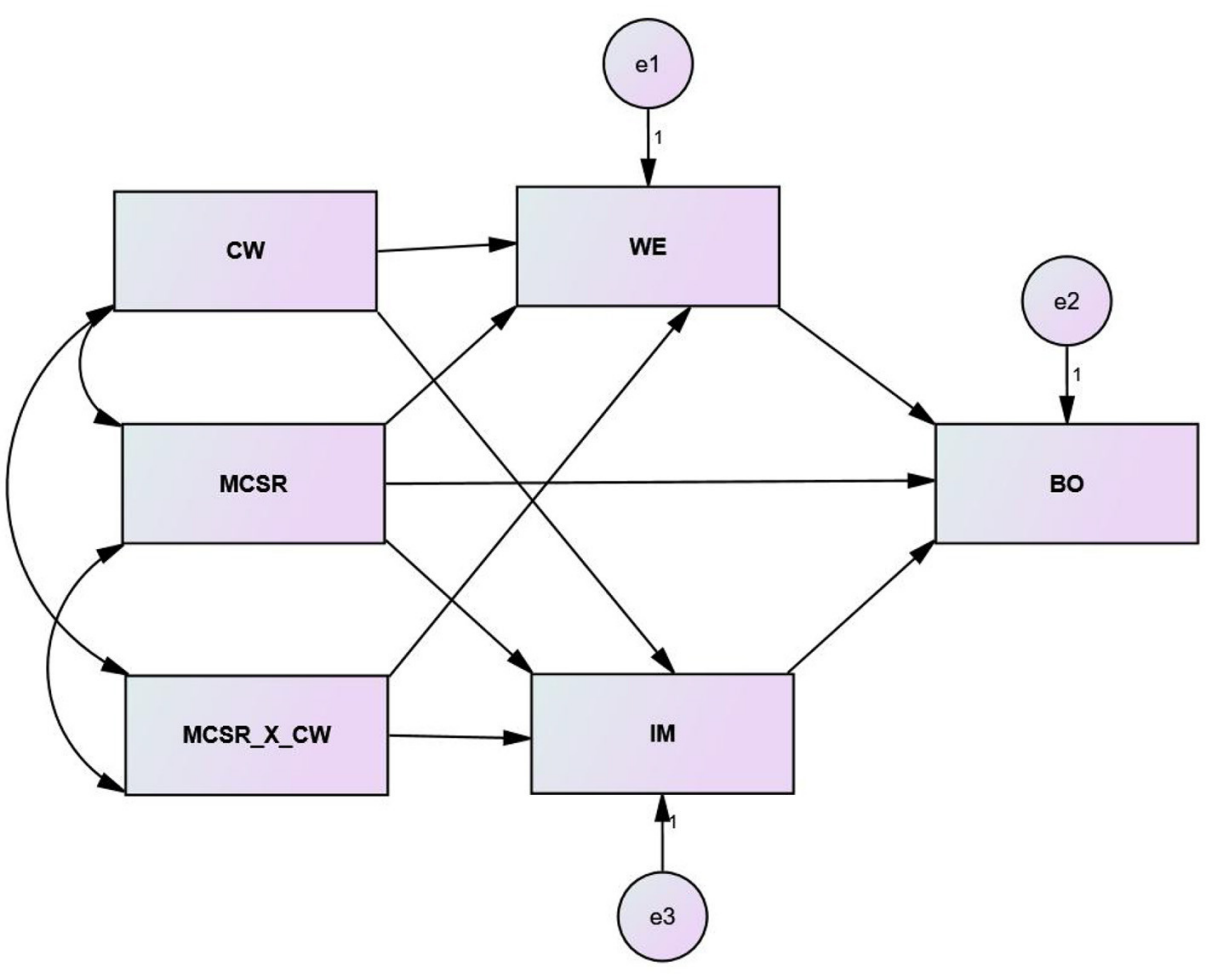
The hypothesized structural model.

The hypothesized theoretical framework is given in Figure 1.

## 3. Methodology

### 3.1. Unit of analysis, sample, and procedure

The unit of analysis in this study was the individual healthcare employees working in different hospitals in Pakistan. For data collection, we targeted two metropolitan cities like, Lahore and Karachi. These cities are the provincial capitals of Punjab and Sindh provinces respectively. Although many public and private hospitals exist in Lahore and Karachi, these hospitals have to attend a huge patient traffic regularly. Therefore, most hospitals face an overburdened situation because hospital staff has to work long hours. Long and irregular working hours are not the only issues for HCW in Pakistan, but low nurse-to-patient and clinician-to-patient ratios also put extra pressure on employees. This is why the epic of BO is more evident in this segment compared to others.

We contacted the administration of different hospitals to verify their CSR engagement. It was realized that large hospitals were carrying out different CSR-related activities with a focus on employees and other stakeholders. We requested different hospitals to facilitate us in this data collection activity. A total of seven hospitals responded to us positively in this regard (five from Lahore and two from Karachi). We invited HCW from different departments to partake in this volunteer activity. Specifically, the data were gathered in a period of 2 months (January–March 2022).

### 3.2. Data design

Specifically, there are four major research designs in the field of quantitative research comprising descriptive, relational, explanatory and experimental. Because the basic purpose of this study was to investigate how different variables influence BO of HCW in a specific context, therefore, this study opted for an explanatory research design. This particular study design basically emphasizes establishing causal relationship between the variables under investigation. Additionally, as this study states various hypotheses, hence it follows deductive reasoning. The real-time data was primary and cross-sectional in nature. Different HCW were randomly invited to partake in this data collection activity.

### 3.3. Data collection instrument and measures

We provided the respondents in this survey with a printed version of a self-administered questionnaire for which different question statements were adapted from different published and reliable sources. Specifically, we considered already available published scales in this survey because such scales have their pre-known validity and reliability. Moreover, field experts were invited to comment on the suitability of these adapted statements with respect to this survey's objective (91–93). After the experts assessed the questionnaire, the finalized version of the questionnaire was prepared, including two major portions. In the first portion, we invited the participants to share their socio-demographic information, whereas, in the next section, the respondents were asked to provide variable-related information on a five point Likert scale. To ensure the ethical standards in this data collection activity, we observed the principal guidelines of the Helsinki Declaration (94–97).

The independent variable MCSR was measured by using a 12-item scale developed by Turker (98), which is a famous scale to measure CSR perceptions of employees. The original scale consisted of a total of 17 items. However, five items were related to customers and government, which were not our subjects in this study. Therefore, we considered 12 items from this scale. Among these 12 items, half (six items) were related to the general employees' CSR perception of their organization, whereas the other half (remaining six) of this scale were related to CSR activities performed by an organization for the betterment of employees (Illustrated items included “This hospital participates in activities that aim to protect and improve the quality of the natural environment (general CSR)” and “This hospital implements flexible policies to provide a good work environment and life balance for its employees (employee related CSR)”.

The dependent variable BO was measured with the help of 7-items which were adapted from Kristensen et al. (99). This scale is a reliable scale to measure employees' BO perceptions in an organization (Illustrated items were “I feel worn out at the end of the working day” and “I am exhausted in the morning at the thought of another day at work”). To measure WE, we used UWES-3 (a 3-item scale) developed by Schaufeli et al. (100). This is basically a short version of UWES-9 by the same authors (Illustrated statement from this scale was “I am enthusiastic about my job”). Similarly, we measured IM by using 5-items from the study of Tierney, Farmer, and Graen (101). The illustrated item was “I enjoy finding solutions to complex problems”.

Lastly, 3-items to measure CW were borrowed from Lilius et al. (102). A sample item from this scale was “How frequently do you experience compassion on the job?” For the CW scale, we used a five-point Likert scale ranging from 1 = never to 5 = very often. All variables' inter-item consistency (α) was significant (MCSR = 0.89, BO = 0.91, WE = 0.75, IM = 0.88, CW = 0.83).

Because this study used the self-reported method, there may be an issue of common method variance (CMV) which is a critical issue in survey research studies (like this study). We took different procedural measures and performed different tests to detect this issue of CMV in our dataset. At a procedural level, we collected data in three waves by separating independent and dependent variables. Each wave was managed at an interval of 2 weeks. Similarly, we assured respondents' anonymity and used simple wording in the statements of all questions. Likewise, the items of a variable were randomly scattered in the questionnaire. Empirically, we performed a single confirmatory factor analysis test which showed a poor model fit, indicating that a one-factor model did not fit with the dataset of this study. Lastly, we also performed a common latent factor (CLF) test to detect CMV in our dataset. For this purpose, two measurement models were developed (actual model vs. CLF-contrasted model). To see if there were significant variations in the regression weights of both models, we compared both models carefully and found that no significant difference (>0.2) in any case existed. This again confirmed that CMV was not a critical issue.

### 3.4. Sample size and data cleaning

We distributed 500 questionnaires initially and received back 358 responses. However, after the data-cleaning phase, we removed 16 responses that were identified as outliers. Similarly, 24 responses were removed due to missing information. Finally, 318 valid responses were considered to proceed with the data analysis. For further detail on data cleaning and outliers, we refer to Tables 1, 2. The socio-demographic statistics revealed that both male and female employees responded to this data collection activity (66% were male). Similarly, the ages of the maximum employees were between 18 and 45 years (around 87%). The experience varied from 1 to 10 (89%).

**Table 1:** Data cleaning summary.

	**Initial sample**	**Received**	**Not-received**	**Deleted**	**Outliers**	**Final response**
	500	358	142	40	16	318
Percentage	-	71.6%	28.4%	11.2%	40.0%	63.6%

**Table 2:** Observations identified as outliers.

**Response number**	**Mahalanobis d-squared**	**p1**	**p2**
228	12.397	0.006	0.040
52	12.096	0.007	0.022
107	12.096	0.007	0.006
162	12.096	0.007	0.002
218	12.096	0.007	0.000
272	12.096	0.007	0.000
292	12.096	0.007	0.000
19	9.535	0.023	0.049
74	9.535	0.023	0.024
129	9.535	0.023	0.011
185	9.535	0.023	0.005
239	9.535	0.023	0.002
43	8.577	0.035	0.044
98	8.577	0.035	0.024
153	8.577	0.035	0.013
209	8.577	0.035	0.006

### 3.5. Initial statistical analysis

Before structural analysis, we performed different statistical tests to confirm the validity and reliability. In this regard, we independently tested the convergent validity of this study's variables. In doing so, firstly, we observed the standardized factor loadings of all variable's items (λ). It was observed that the λ values were significant in all cases (>0.5 or ideally >0.7) (22, 103, 104). These λ values were then considered to estimate the average variance extracted (AVE) for all five variables. The below formula (Equation-1) was used to calculate AVEs.


$$\begin{array}{l} \text{AVE}\;=\;\frac{\sum_{\overset{•}{\text{i}}=1}^{\text{k}}\lambda_{\text{i}}^{2}}{\sum_{\overset{•}{i}=1}^{\text{k}}\lambda_{\text{i}}^{2}+\sum_{\text{i}=1}^{\text{k}}.\text{var}(\varepsilon \text{i})} \end{array}$$

AVE values were significant in all cases (>0.5), confirming that the convergent validity was significant for all variables. Specifically, the AVEs varied from 0.56 (IM) to 0.72 (WE).

We also performed a reliability test, especially a composite reliability test, to confirm if the items of a variable were consistent. For such calculations, we used the below equation.


Composite reliability = ((∑λi)2)/(∑λi)2+∑var(εi))

The statistical results showed that the composite reliability was statistically significant in all cases as all values were >0.7 (105–107). Precisely, composite reliability values were between 0.86 (IM) to 0.94 (MCSR). For further detail, we refer to below Table 3.

**Table 3:** Validity and reliability.

	**λ**	**λ^2^**	**E-variance**
**MCSR**			
	0.72	0.52	0.48
	0.79	0.62	0.38
	0.70	0.49	0.51
AVE = 0.59	0.76	0.58	0.42
CR = 0.94	0.83	0.69	0.31
∑λ^2^ = 7.07	0.88	0.77	0.23
Items = 12	0.70	0.49	0.51
	0.72	0.52	0.48
	0.73	0.53	0.47
	0.82	0.67	0.33
	0.74	0.55	0.45
	0.80	0.64	0.36
**BO**			
	0.84	0.71	0.29
	0.76	0.58	0.42
AVE = 0.65	0.73	0.53	0.47
CR = 0.93	0.84	0.71	0.29
∑λ^2^ = 4.58	0.88	0.77	0.23
Items = 7	0.79	0.624	0.38
	0.81	0.66	0.34
**WE**			
AVE = 0.72	0.76	0.58	0.42
CR = 0.88	0.92	0.85	0.15
∑λ^2^ = 2.16	0.86	0.74	0.26
Items = 3	-	-	-
**IM**			
AVE = 0.56	0.70	0.49	0.51
CR = 0.86	0.72	0.52	0.48
∑λ^2^ = 2.80	0.76	0.58	0.42
Items = 5	0.78	0.61	0.39
	0.78	0.61	0.39
**CW**			
AVE = 0.68	0.79	0.62	0.38
CR = 0.87	0.83	0.69	0.31
∑λ^2^ = 2.05	0.86	0.74	0.26
Items = 3	-	-	-

λ, Item loadings; CR, composite reliability; ∑λ^2^, sum of square of item loadings; E-Variance, error variance.

## 4. Results

### 4.1. Model fitness

We tested whether there was a reasonable fit between this study's theoretical model and statistical data. To do this, we developed four different measurement models (see Table 4), among which model 1 was this study's actual model (five-factor), and the rest of the three models were the alternate models. To decide which model best fits the dataset of this study, we checked different model fit indices values (especially GFI, TLI, IFI, CFI, etc.) and 2/df values along with RMSEA values. This exercise revealed that model-1 was superior in all respects (GFI = 0.94, TLI = 0.95, IFI = 0.96, CFI = 0.96, RMSEA = 0.052, and 2/df = 2.19), indicating that there was an excellent fit betwixt theory and data. The rest of the alternate models produced mixed results (for example, model- 4 produced poor results in all respects, and model 2 showed some better results but not excellent).

**Table 4:** Model fitness.

**Model**	**Composition**	***χ^2^/df*** **(< 3)**	**Δχ^2^*/df*** ***-***	**RMSEA** **(< 0.08)**	**GFI** **(>0.9)**	**TLI** **(>0.9)**	**IFI** **(>0.9)**	**CFI** **(>0.9)**
1	MCSR, BO, WE, IM, CW	2.19	_	0.052	0.94	0.95	0.96	0.96
2	MCSR + CW + WE, IM, BO	5.03	3.54	0.067	0.74	0.76	0.78	0.76
3	MCSR + CW, WE + IM + BO	7.84	0.94	0.152	0.49	0.48	0.50	0.50
4	MCSR + BO + WE + IM + CW	8.42	2.19	0.200	0.42	0.44	0.44	0.43

### 4.2. Model fitness

We assessed correlations (*r*) between different variables pairs. Some pairs showed positive association (for example, MCSR < = >WE = 0.42) whereas, some cases were negatively correlated (for example, MCSR < = >BO = −0.50). However, all cases showed significant values of *r* (*p* < 0.05, 0.01). Equally important to note is the fact that no *r* value was between the critical range (>0.8). Table 5 summarizes the results of correlations, discriminant validity (bold values), mean and standard deviation values.

**Table 5:** Correlations and discriminant validity.

**Construct**	**1**	**2**	**3**	**4**	**5**
1. MCSR	**0.77**	–0.50	0.42	0.55	0.28
2. BO	(2.88, 0.42)	**0.81**	−0.48	−0.45	−0.34
3. WE		(2.95, 0.44)	**0.85**	0.47	0.37
4. IM			(3.28, 0.62)	**0.75**	0.36
5. CW				(2.99, 0.47)	**0.83**
					(3.12, 0.57)

Values in parenthesis = Mean and standard deviation; bold values = discriminant validity.

*p* < 0.001.

### 4.3. Main analysis

After performing the above stats in the preliminary analysis, we performed the main analysis to validate the hypothesized relationships. To do so, a structural model was developed in AMOS. Prior to developing this structural model, we mean-centered MCSR and CW variables. Similarly, a bootstrapping sample (5,000) was inserted to see the mediation and moderation effects. Moreover, the PROCESS Macro tool by Hayes (108) was also considered to calculate different equations in AMOS by using user-defined estimand. Precisely the model 7 equation guidelines were observed for this structural analysis. An interaction term was also developed by multiplying the mean scores of MCSR and CW (MCSR_X_CW). The results of this structural analysis have been summarized in Table 6. According to the results the direct effect (H1, H2, H4) were all significant (H1 = −0.52, H2 = 0.40, H4 = 0.58, *p* < 0.05 with non-zero CI-values).

**Table 6:** Hypotheses results.

**Hypotheses**	**Estimates (SE)**	***t/z***	***p-*value**	**CI**
(MCSR → BO)	−0.52 (0.059)	−8.81	^***^	−0.72, −0.49
(MCSR → WE)	0.40 (0.066)	6.06	^***^	0.33, 0.56
(MCSR → IM)	0.58 (0.069)	8.40	^***^	0.29, 0.66
Indirect effect				
(MCSR → WE → BO)	−0.37 (0.048)	−7.71	^***^	−0.48, −0.25
MCSR → IM → BO	−0.40 (0.053)	−7.55	^***^	−0.24, −0.48
The conditional indirect effect of CW betwixt MCSR → WE → BO	−0.30 (0.033)	−9.09	^***^	−0.37, −0.22
The conditional indirect effect of CW betwixt MCSR → IM → BO	−0.33 (0.029)	−11.4	^***^	−0.39, −0.29

CI = 95% confidence interval with lower and upper limits.

^^***^^p < 0.001.

Next, we investigated the structural model for indirect effects (H3, H5). The results confirmed that there are significant mediation effects between MCSR and BO *via* WE and IM (H3 = −0.37, and H5 = −0.40, *p* < 0.05 with non-zero CI-values). Hence H3 and H5 were statistically accepted. Similarly, the conditional effects of CW between MCSR and WE and between MCSR and IM were also significant. Precisely, we tested these effects at three different values of CW (± 1 SD and at mean value). These results confirmed that CW significantly moderates between MCSR → WE and between MCSR → IM. Hence it was established that there is a significant buffering effect in the mediated relationships between MCSR and BO *via* WE (−0.30, *p* < 0.05) and *via* IM (−0.33, *p* < 0.05). Thereby, it was established that H6 and H7 were significant.

## 5. Discussion

The major objectives specified at the beginning of this study can now be discussed in detail in light of statistical evidence. For example, the empirical investigation supports our theoretical assumption that MCSR significantly predicts employees' BO. Generally, employees show the symptoms of BO in an organization when they feel any situation of losing, depletion or lack of valued resources, for example, employee acknowledgment for accomplishing some important task, life meaning or purpose, supporting organizational environment, and a working place where they feel less value conflicts. In this respect, the ethical engagement of an organization creates a positive work environment in which employees are expected to happily perform different organizational tasks in response to the caring attitude of their ethical organization. Additionally, both the general CSR perceptions in employees' minds about their organization and employee-focused CSR activities are of profound importance to improving mental health and employee wellness. Employees with an improved mental health level are more resilient and energetic to face the risk of BO. Therefore, they are less overwhelmed to be burned-out while fixing a difficult situation in a workplace. The contextual support by an ethical organization to the employees provides them with added resources to fight against BO. Therefore, employees feel less resource constraints in an ethical organization, thereby reducing the chances of burned-out. This finding also receives support from the extant literature (33, 34).

Another important objective of carrying out this study was to explain the underlying mechanism betwixt MCSR-BO relationship with the help of two mediators (WE, IM) and a moderator (CW). We feel it was important to understand the underlying mechanism of how and why MCSR reduces employees' BO in an organization. Previous literature mentions that MCSR relates to different employee outcomes, including BO, however, the underlying mechanisms to better explain the phenomenon of BO were not discussed. To this end, our study shows that MCSR activities of an organization improve the engagement level of employees in an organization. Various steps taken by an ethical organization for the welfare of employees are well observed by the employees who improve their commitment, motivation and engagement. Not only are the employees' internal CSR initiatives appreciated by the employees, but the general CSR engagement of an organization for the external community and stakeholders also leaves a pleasant impression on employees, and they feel pride in being the workers of an ethical organization. As an antecedent of CSR, this feeling of pride improves their engagement level. Engaged employees are less prone to be burned-out soon while facing an uncertain situation. Moreover, engaged employees do not build this feeling that they have insufficient resources to perform their job. Hence they are less likely to face the risk of BO (57, 58). Hence this study confirms the mediating effect of WE betwixt MCSR and BO.

Another important underlying mechanism that explains why MCSR reduces BO among employees is the consideration of IM as a mediator between MCSR and BO. To this end, there is increasing evidence in the current literature that individuals with a higher level of IM concentrate more on completing their tasks. Indeed, IM is assumed as one of the valued personal resources that resist different stressors, including BO. Employees with an improved level of IM have increased personal resources and are expected to be more protected against the negative effects of BO. An organization's engagement in MCSR-related activities improves the IM level of employees because an ethical organization shows a genuine concern for all stakeholders, including employees, which ultimately improves their IM level. A socially responsible organization's ethical concern for employees' welfare urges them to respond positively to their organization. Therefore, employees show an extra level of energy and vigor to complete their job tasks, thereby, they face less risk of BO. This finding is in line with previous studies as well (79, 80). Hence, our study confirms the mediating effect of IM betwixt MCSR and BO.

Lastly, our results support the theoretical statements of H6 and H7 by verifying that CW provides a buffering effect by acting as a moderator between MCSR-WE and MCSR-IM. Especially in a healthcare context, CW s assumed to be the foundation. Indeed CW relates to the concern, suffering, and distress of patients and their families by taking different actions for their relief from a disease or illness. In a healthcare environment, CW is all about listening, respecting, and empathizing with the pain and suffering of patients. Compassion and kindness are not only beneficial for the receivers, but they have an advantage for the providers and are the source of motivation for individuals to join healthcare services. In the presence of CW, healthcare employees are more effective with high morale and show greater motivation for patient care and safety. Conceptually, CW helps employees in developing an environment in which they prefer the collective benefit of others which is also a concern of CSR. Therefore, CW and CSR share the same concern, which is the collective benefit of others. The ethical commitment of an organization provides a context for the workers to build a higher level of CW.

Compassionate employees are better engaged and intrinsically motivated to complete their job tasks because their enhanced level of CW creates positive emotions. At the same time, compassionate workers look for the collective benefit of others. Hence CW provides a buffering effect for enhancing the WE and IM among employees in an ethical organization. Previous literature also indicates that compassionate people show a higher level of engagement and motivation to complete their job tasks (88, 89). Therefore, this study confirms the moderating effect of CW between the mediated relationship of MCSR and BO *via* WE and IM.

Lastly, as we mentioned earlier that all contacted hospitals did not respond us positively. There may be different reasons why some hospitals did not participate in this study. For example, tight schedules of the staff, procedural delays, administrative issues, and policy and security issues may be some reasons due to which some hospitals were reluctant to partake in this data collection activity.

### 5.1. Contributions in theory

This study contributes to the existing body of knowledge in three ways. Firstly, this study is one of the fewest studies which relate MCSR with negative employee outcomes, especially from the perspective of BO in the healthcare sector. BO is a critical issue in healthcare management that not only affects the mental health of HCW, but is also a concern of public health because HCW with poor mental health is likely to undermine the quality of patient healthcare delivery, which ultimately undermines the overall public. With an increasing rate of BO in a healthcare system, the mental health of HCW worsens, undermining the public's ability to access healthcare treatment, making it harder for a nation to deal with a public health emergency, increasing health disparities, and ultimately creating a public health crisis. Although BO was previously debated in a healthcare context, however, previously MCSR was not discussed in relation to BO, especially in a healthcare context. Specifically, this research extends the theoretical framework by Nazir and Islam (63), who showed how the mechanism between CSR, compassion, and WE works, however, these authors were unable to relate how better WE can reduce BO of employees. Another important extension that this study makes to the existing body of knowledge is the work by Svergun and Fairlie (109), who investigated the direct impact of CSR on different employee outcomes, for example, depression and turnover intentions. However, these scholars missed highlighting the underlying mechanism of how and why CSR is an important predictor of negative employee outcomes.

Secondly, previously the phenomenon of BO, especially in a healthcare context, was mainly examined in developed countries, leaving the terrain of developing countries unattended. Our argument here is that the phenomenon of employee BO presents a worse situation in developing economies due to the poor social and infrastructural support compared to developed countries. Moreover, the healthcare resources in most developing countries are scarce, making it more challenging for healthcare administration to pursue different organizational management strategies, thereby creating more demanding workplace situations and increasing the risk of BO. Hence, studies conducted in developed countries may not reflect the case of developing countries.

Thirdly, this study tends to contribute to the existing body of knowledge by proposing a robust model to explain the MCSR-BO relationship because the model proposed in this study enriches the existing body of knowledge to understand the underlying mechanism between MCSR and BO with the help of mediators and moderator. Previously, no such relationships were investigated in a unified model.

### 5.2. Contributions to the field

This study significantly contributes to healthcare administration and management, especially how to deal with the epic of BO in this sector. Given that BO has been a critical issue in the healthcare sector, our study shows that the CSR orientation of an ethical hospital organization can significantly reduce the risk of BO among employees. Considering the rising number of employees' BO in the healthcare sector worldwide and considering the limited financial resources available for the health sector, an effective approach is required to mitigate BO syndrome among HCW. Even from the perspective of public health, a hospital's CSR strategies influence public health by helping employees to meager the effect of negative outcomes such as stress and depression that ultimately determine BO. Healthcare employees with better mental and physical health, as an outcome of MCSR, are expected not to undermine the quality of health delivery to the citizens of a country, thereby contributing positively toward an improved level of public health. Therefore, an employer may fix BO in healthcare by carefully planning and executing different MCSR plans.

In addition, the MCR activities of a hospital organization not only have a direct negative relationship with employees' BO, such activities also improve positive employee psychology by enriching the engagement level and motivation level of employees, which provides a further explanation by providing mediation effect betwixt MCSR and BO. MCSR also improves the compassion level of workers, which then provides a buffering effect to reduce the BO level of employees to a further level. Therefore, if a hospital administration wants to improve the BO syndrome among employees, it should realize the true potential of MCSR activities in reducing employees' BO in a healthcare context.

### 5.3. Limitations and future suggestions

Although this study offers different implications on theoretical and practical landscapes, it still faces some potential limitations that we consider important to highlight, along with different suggestions. First, this study collected the data only from Lahore and Karachi. Though these cities were important from a data collection perspective because they constitute a very large number of hospitals (both public and private), we still feel the geographic concentration of this study may limit its generalizability. Therefore, we recommend including more cities from other provinces. Sample representativeness is another potential issue, although the data were collected from healthcare employees. However, due to different policy reasons and security parameters, most hospitals were only kind enough to let us allow entering their premises for data collection, but no hospital shared any stats on hospital employees, departments, etc. Due to this difficulty, deciding on sample representativeness was not possible. In the future, we suggest incorporating this limitation by getting such stats on employees (if possible). Another potential issue with this study was the non-probability sampling technique which limits the significance of causal relationships. Therefore, in future studies, we suggest pursuing a probability sampling method to have a better causality claim. Lastly, as cultures may be important in leadership studies, we feel in similar cultures (India, for example), our study may reflect similar results, however, a due consideration is requested before interpreting our results in different cultures (western culture, for example).

## 6. Conclusion

The healthcare administration needs to realize that with the rise of BO, the mental health crisis worsens, ultimately affecting employees' capability to deliver superior health to the citizens and patients, thereby creating a public health concern. Especially in developing regions, where health disparities are already huge, the issue of BO is more critical due to poor infrastructure and insufficient resources. To better fix the issue of employees' BO in healthcare, a collective and system-based solution is required for which carefully planned MCSR strategies could be a way forward for a hospital administration. A hospital's ethical context always gives its employees hope for a positive present and an excellent future, which infuses positive emotions, for example, in the form of WE and IM, among employees, and they show better energy levels in completing their job tasks. Specifically, the MCSR orientation of a hospital organization gives this hope to the employees that their organization is a caring one which not only promotes the interest of shareholders, but it also considers the interest of employees equally important. This process ultimately infuses positive energy among employees and reduce BO as shown in the empirical analysis of this study that the manifestation of MCSR significantly reduces BO risk among healthcare employees (beta = −0.52). For a better outcome, we suggest hospital administration closely align CSR strategy with employee welfare plans so that employee can clearly see a pleasant future where their dedication isn't taken for granted and where their health, safety, and wellbeing is as much a priority as the wellbeing of the people and communities in their care. Similarly, we also suggest hospital administration align employees' training and CSR programs that could enhance their engagement and motivation level as well as their compassion level. Employees with an improved level of IM, WE and compassion can be well placed to fight the epic of BO in healthcare again.

## Data availability statement

The raw data supporting the conclusions of this article will be made available by the authors, without undue reservation.

## Ethics statement

The studies involving human participants were reviewed and approved by Xiamen University and the Institutional Review Board of the Pakistan Kidney and Liver Institute and Research Centre. Respondents provided their written informed consent to participate in this study.

## Author contributions

All authors listed have made a substantial, direct, and intellectual contribution to the work and approved it for publication.

## References

[1] Barnes EL Ketwaroo GA Shields HM. Scope of burnout among young Gastroenterologists and practical solutions from gastroenterology and other disciplines. Dig Dis Sci. (2019) 64:302–6. 10.1007/s10620-018-5443-3 30607687

[2] Embriaco N Papazian L Kentish-Barnes N Pochard F Azoulay E. Burnout syndrome among critical care healthcare workers. Curr Opin Crit Care. (2007) 13:482–8. 10.1097/MCC.0b013e3282efd28a 17762223

[3] De Hert S. Burnout in healthcare workers: prevalence, impact and preventative strategies. Local Reg Anesth. (2020) 13:171. 10.2147/LRA.S240564 33149664 PMC7604257

[4] Kasemy ZA Abd-Ellatif EE Abdel Latif AA Bahgat NM Shereda HMA Shattla SI. Prevalence of Workaholism among Egyptian healthcare workers with assessment of its relation to quality of life, mental health and burnout. Front Public Health. (2020) 8:581373. 10.3389/fpubh.2020.581373 33324599 PMC7725873

[5] Dijxhoorn A-FQ Brom L van der Linden YM Leget C Raijmakers NJ. Prevalence of burnout in healthcare professionals providing palliative care and the effect of interventions to reduce symptoms: a systematic literature review. Palliat Med. (2021) 35:6–26. 10.1177/0269216320956825 33063609

[6] Maslach C Schaufeli WB Leiter MP. Job burnout. Annu Rev Psychol. (2001) 52:397–422. 10.1146/annurev.psych.52.1.397 11148311

[7] Cotel A Golu F Pantea Stoian A Dimitriu M Socea B Cirstoveanu C. Predictors of burnout in healthcare workers during the COVID-19 pandemic. Healthcare. (2021) 9:304. 10.3390/healthcare9030304 33803286 PMC8001536

[8] Dimitriu MC Pantea-Stoian A Smaranda AC Nica AA Carap AC Constantin VD. Burnout syndrome in Romanian medical residents in time of the COVID-19 pandemic. Med Hypotheses. (2020) 144:109972. 10.1016/j.mehy.2020.109972 32531540 PMC7276114

[9] National Academy of Medicine. Taking Action Against Clinician Burnout: A Systems Approach to Professional Wellbeing. (2019). Available online at: https://nam.edu/wp-content/uploads/2019/10/CR-report-highlights-brief-final.pdf (accessed October 1, 2019). 31940160

[10] Yao H Wang J Bo Q Li M. Sadder but wiser: the role of SARS imprinting and firms' recovery during the COVID-19 pandemic. Front Psychol. (2022) 13:917337. 10.3389/fpsyg.2022.917337 35756304 PMC9226627

[11] Fabio P Stefania S Elisabetta T Thi TCN Iolanda G. Public health and burnout: a survey on lifestyle changes among workers in the healthcare sector. Acta Bio Medica: Atenei Parmensis. (2019) 90:24. 10.23750/abm.v90i1.7626 30889151 PMC6502147

[12] Iwanicki EF Schwab RL. A cross validation study of the Maslach Burnout Inventory. Educ Psychol Meas. (1981) 41:1167–74. 10.1177/001316448104100425

[13] Maslach C Jackson SE Leiter MP. (1997). Maslach Burnout Inventory. Scarecrow Education.

[14] World Health Organization. Burnout an “occupational phenomenon”: International Classification of Diseases. (2019). Available online at: https://www.who.int/news/item/28-05-2019-burn-out-an-occupational-phenomenon-international-classification-of-diseases (accessed May 28, 2019).

[15] Jennifer M. (2019). Burnout Is About Your Workplace, Not Your People. Available online at: https://hbr.org/2019/12/burnout-is-about-your-workplace-not-your-people (accessed December 11, 2019).

[16] Parveen M Adeinat I. Transformational leadership: does it really decrease work-related stress? Leadersh Organ Dev J. (2019) 40:860–76. 10.1108/LODJ-01-2019-0023

[17] Tafvelin S Nielsen K von Thiele Schwarz U Stenling A. Leading well is a matter of resources: leader vigour and peer support augments the relationship between transformational leadership and burnout. Work & Stress. (2019) 33:156–72. 10.1080/02678373.2018.1513961

[18] Dal Corso L De Carlo A Carluccio F Colledani D Falco A. Employee burnout and positive dimensions of wellbeing: A latent workplace spirituality profile analysis. PLoS ONE. (2020) 15:e0242267. 10.1371/journal.pone.0242267 33201895 PMC7671502

[19] Bakker AB de Vries JD. Job Demands–Resources theory and self-regulation: new explanations and remedies for job burnout. Anxiety, Stress, Coping. (2021) 34:1–21. 10.1080/10615806.2020.1797695 32856957

[20] Wang Q Wang C. Reducing turnover intention: perceived organizational support for frontline employees. Front Bus Res China. (2020) 14:1–16. 10.1186/s11782-020-00074-6

[21] Shao J Zhang T Wang H Tian Y. Corporate social responsibility and consumer emotional marketing in big data era: a mini literature review. Front Psychol. (2022) 13:919601. 10.3389/fpsyg.2022.919601 35693492 PMC9178265

[22] Xu L Mohammad SJ Nawaz N Samad S Ahmad N Comite U. The role of CSR for De-carbonization of hospitality sector through employees: a leadership perspective. Sustainability. (2022) 14:5365. 10.3390/su14095365

[23] Ahmad N Ullah Z AlDhaen E Han H Scholz M. A CSR perspective to foster employee creativity in the banking sector: The role of work engagement and psychological safety. J Retail Consum Serv. (2022) 67:102968. 10.1016/j.jretconser.2022.102968

[24] Aguinis H. Organizational responsibility: Doing good and doing well. In: Zedeck S, editor. APA Handbook of Industrial and Organizational Psychology, Vol 3: Maintaining, Expanding, and Contracting the Organization. Washington, DC, US: American Psychological Association. (2011). 10.1037/12171-024

[25] Kumasey AS Delle E Agbemabiase GC. Benefits of Promoting Micro-Level Corporate Social Responsibility for Emerging Economies Responsible Management in Emerging Markets. New York: Springer. (2021) p. 37–61. 10.1007/978-3-030-76563-7_2

[26] Low MP Spong H. Predicting employee engagement with micro-level corporate social responsibility (CSR) practices in the public accounting firms. Soc Responsib J. (2022) 18:266–92. 10.1108/SRJ-07-2020-0300

[27] Id Bouichou S Wang L Zulfiqar S. How perceived corporate social responsibility raises employees' creative behaviors based on appraisal theory of emotion: the serial mediation model. Front Psychol. (2022) 13:865007. 10.3389/fpsyg.2022.865007 35432100 PMC9006776

[28] Mahmud A Ding D Ali Z. An investigation of employee perception of micro-corporate social responsibility and societal behavior: a moderated-mediated model. International J Emerg Mark. (2021). 10.1108/IJOEM-02-2021-0266. [Epub ahead of print].

[29] Ahmad N Ullah Z AlDhaen E Han H Araya-Castillo L Ariza-Montes A. Fostering hotel-employee creativity through micro-level corporate social responsibility: a social identity theory perspective. Front Psychol. (2022) 13. 10.3389/fpsyg.2022.853125 35572307 PMC9093142

[30] Srivastava RV Tang T. The Matthew effect in talent management strategy: reducing exhaustion, increasing satisfaction, and inspiring commission among boundary spanning employees. J Bus Ind Mark. (2022) 37:477–96. 10.1108/JBIM-06-2020-0296

[31] Cheng Y Wang Y Pan F. The impact of CSR perceptions on employees' turnover intention during the COVID-19 crisis in China. Int J Environ Res Public Health. (2022) 19:8297. 10.3390/ijerph19148297 35886148 PMC9318882

[32] Adu-Gyamfi M He Z Nyame G Boahen S Frempong MF. Effects of internal CSR activities on social performance: the employee perspective. Sustainability. (2021) 13:6235. 10.3390/su13116235

[33] Ramdhan RM Kisahwan D Winarno A Hermana D. Internal corporate social responsibility as a microfoundation of employee well-being and job performance. Sustainability. (2022) 14:9065. 10.3390/su14159065

[34] Rezaee N Babakhani N Bagheri N. The relationship between psychological capital and burnout with mediated the social responsibility perception in nurses. J Couns Psychol. (2021) 10:50–61.

[35] Glavas A. Corporate social responsibility and employee engagement: enabling employees to employ more of their whole selves at work. Front Psychol. (2016) 796. 10.3389/fpsyg.2016.00796 27303352 PMC4886691

[36] Alfes K Truss C Soane EC Rees C Gatenby M. The relationship between line manager behavior, perceived HRM practices, and individual performance: examining the mediating role of engagement. Hum Resour Manage. (2013) 52:839–59. 10.1002/hrm.21512

[37] Purc E Laguna M. Personal values and innovative behavior of employees. Front Psychol. (2019) 10:865. 10.3389/fpsyg.2019.00865 31057470 PMC6482311

[38] Todd D. Leading Health Care Organizations Declare Physician Burnout as ‘Public Health Crisis'. (2019). Available online at: https://www.hsph.harvard.edu/news/press-releases/leading-health-care-organizations-declare-physician-burnout-as-public-health-crisis/ (accessed January 17, 2019).

[39] Wang J Wang W Laureys S Di H. Burnout syndrome in healthcare professionals who care for patients with prolonged disorders of consciousness: a cross-sectional survey. BMC Health Serv Res. (2020) 20:1–10. 10.1186/s12913-020-05694-5 32894132 PMC7487695

[40] Bangdiwala SI Fonn S Okoye O Tollman S. Workforce resources for health in developing countries. Public Health Rev. (2010) 32:296–318. 10.1007/BF03391604

[41] Von Elm E Altman DG Egger M Pocock SJ Gøtzsche PC Vandenbroucke JP. The strengthening the reporting of observational studies in epidemiology (strobe) statement: guidelines for reporting observational studies. J Clin Epidemiol. (2008) 61:344–349. 10.1016/j.jclinepi.2007.11.008 18313558

[42] Alvaro C Lyons RF Warner G Hobfoll SE Martens PJ Labonté R. Conservation of resources theory and research use in health systems. Implementat Sci. (2010) 5:1–20. 10.1186/1748-5908-5-79 20961445 PMC2978118

[43] Penney LM Hunter EM Perry SJ. Personality and counterproductive work behaviour: Using conservation of resources theory to narrow the profile of deviant employees. J Occup Organ Psychol. (2011) 84:58–77. 10.1111/j.2044-8325.2010.02007.x

[44] Neveu JP. Jailed resources: Conservation of resources theory as applied to burnout among prison guards. J Organ Behav. (2007) 28:21–42. 10.1002/job.393

[45] Prapanjaroensin A Patrician PA Vance DE. Conservation of resources theory in nurse burnout and patient safety. J Adv Nurs. (2017) 73:2558–65. 10.1111/jan.13348 28543427

[46] Hobfoll SE. Conservation of resources: a new attempt at conceptualizing stress. Am Psychol. (1989) 44:513. 10.1037/0003-066X.44.3.513 2648906

[47] Lin C-P Liu M-L. Examining the effects of corporate social responsibility and ethical leadership on turnover intention. Personnel Rev. (2017) 46:526–50. 10.1108/PR-11-2015-0293

[48] Hobfoll SE. The influence of culture, community, and the nested-self in the stress process: advancing conservation of resources theory. Appl Psychol. (2001) 50:337–421. 10.1111/1464-0597.00062

[49] Alarcon GM Edwards JM Menke LE. Student burnout and engagement: a test of the conservation of resources theory. J Psychol. (2011) 145:211–27. 10.1080/00223980.2011.555432 21560805

[50] Hobfoll SE Freedy J. Conservation of resources: a general stress theory applied to burnout Professional burnout. London, United Kingdom: Routledge. (2017) p. 115–129. 10.4324/9781315227979-9

[51] Palmer C Souza GI Laray E Viana V Hall A. Participatory policies and intrinsic motivation to conserve forest commons. NatSustainability. (2020) 3:620–7. 10.1038/s41893-020-0531-8

[52] Shirom A. Feeling vigorous at work? the construct of vigor and the study of positive affect in organizations. In: Perrewe PL, Ganster DC, editors. Emotional and Physiological Processes and Positive Intervention Strategies. United Kingdom: Emerald Group Publishing Limited. (2003) p. 135–164. 10.1016/S1479-3555(03)03004-X

[53] Van Steenbergen EF van der Ven C Peeters MC Taris TW. Transitioning towards new ways of working: do job demands, job resources, burnout, and engagement change? Psychol Rep. (2018) 121:736–66. 10.1177/0033294117740134 29298562

[54] Mäkikangas A Leiter MP Kinnunen U Feldt T. Profiling development of burnout over eight years: relation with job demands and resources. Eur J Work Organ. (2021) 30:720–31. 10.1080/1359432X.2020.1790651

[55] Leitão J Pereira D Gonçalves Â. Quality of work life and contribution to productivity: Assessing the moderator effects of burnout syndrome. Int J Environ Res Public Health. (2021) 18:2425. 10.3390/ijerph18052425 33801326 PMC7967557

[56] Kennedy DR Clapp P DeLuca JL Filtz TM Kroon L Lamberts JT. Enhancing pharmacy faculty well-being and productivity while reducing burnout. Am J Pharm Educ. (2022) 86:5. 10.5688/ajpe8764 34507961 PMC10159489

[57] Cole MS Walter F Bedeian AG O'Boyle EH. Job burnout and employee engagement: a meta-analytic examination of construct proliferation. J Manage. (2012) 38:1550–81. 10.1177/0149206311415252

[58] Santhanam N Srinivas S. Modeling the impact of employee engagement and happiness on burnout and turnover intention among blue-collar workers at a manufacturing company. Benchmarking. (2020) 27:499–516. 10.1108/BIJ-01-2019-0007

[59] Al-dalahmeh M Khalaf R Obeidat B. The effect of employee engagement on organizational performance *via* the mediating role of job satisfaction: The case of IT employees in Jordanian banking sector. Modern Appl Sci. (2018) 12:17–43. 10.5539/mas.v12n6p17

[60] Antony MR. Paradigm shift in employee engagement–a critical analysis on the drivers of employee engagement. Int J Inf Bus Manag. (2018) 10:32–46.

[61] Jim A Maika L Jennifer R. How Strengths, Wellbeing Engagement Reduce Burnout. (2020). Available online at: https://www.gallup.com/cliftonstrengths/en/312467/strengths-wellbeing-engagement-reduce-burnout.aspx (accessed June 9, 2020).

[62] Bapat S Upadhyay P. Implications of CSR initiatives on employee engagement. Social Responsib J. (2021) 17:149–63. 10.1108/SRJ-05-2018-0120

[63] Nazir O Islam JU. Effect of CSR activities on meaningfulness, compassion, and employee engagement: a sense-making theoretical approach. Int J Hospitality Manag. (2020) 90:102630. 10.1016/j.ijhm.2020.102630

[64] Duthler G Dhanesh GS. The role of corporate social responsibility (CSR) and internal CSR communication in predicting employee engagement: Perspectives from the United Arab Emirates (UAE). Public Relat Rev. (2018) 44:453–62. 10.1016/j.pubrev.2018.04.001

[65] Soni D Mehta P. Manifestation of Internal CSR on Employee Engagement: Mediating Role of Organizational Trust. Indian J Ind Relat. (2020) 55:3.

[66] Jia Y Yan J Liu T Huang J. How does internal and external CSR affect employees' work engagement? Exploring multiple mediation mechanisms and boundary conditions. Int J Environ Res Public Health. (2019) 16:2476. 10.3390/ijerph16142476 31336754 PMC6678673

[67] Hur WM Moon TW Choi WH. When are internal and external corporate social responsibility initiatives amplified? Employee engagement in corporate social responsibility initiatives on prosocial and proactive behaviors. Corp Soc Responsib Environ. (2019) 26:849–58. 10.1002/csr.1725

[68] Ryan RM Deci EL. Intrinsic and extrinsic motivations: Classic definitions and new directions. Contemp Educ Psychol. (2000) 25:54–67. 10.1006/ceps.1999.1020 10620381

[69] Moon T-W Youn N Hur W-M Kim K-M. Does employees' spirituality enhance job performance? The mediating roles of intrinsic motivation and job crafting. Curr Psychol. (2020) 39:1618–34. 10.1007/s12144-018-9864-0

[70] Shin Y Hur W-M Moon TW Lee S. A motivational perspective on job insecurity: Relationships between job insecurity, intrinsic motivation, and performance and behavioral outcomes. Int J Environ Res Public Health. (2019) 16:1812. 10.3390/ijerph16101812 31121833 PMC6571976

[71] Isoard-Gautheur S Ginoux C Gerber M Sarrazin P. The stress–burnout relationship: Examining the moderating effect of physical activity and intrinsic motivation for off-job physical activity. Workplace Health Saf. (2019) 67:350–60. 10.1177/2165079919829497 30873913

[72] Wang E Hu H Mao S Liu H. Intrinsic motivation and turnover intention among geriatric nurses employed in nursing homes: the roles of job burnout and pay satisfaction. Contemp Nurse. (2019) 55:195–210. 10.1080/10376178.2019.1641120 31272295

[73] Cuadrado E Tabernero C Fajardo C Luque B Arenas A Moyano M. Type D personality individuals: exploring the protective role of intrinsic job motivation in burnout. Revista de Psicologí*a del Trabajo y de las Organizaciones*. (2021) 37:133–41. 10.5093/jwop2021a12

[74] Ten Brummelhuis LL Ter Hoeven CL Bakker AB Peper B. Breaking through the loss cycle of burnout: the role of motivation. J Occup Organ Psychol. (2011) 84:268–87. 10.1111/j.2044-8325.2011.02019.x

[75] Farooq Q Liu X Fu P Hao Y. Volunteering sustainability: An advancement in corporate social responsibility conceptualization. Corp Soc Responsib Environ Manag. (2020) 27:2450–64. 10.1002/csr.1893

[76] Hao Y Farooq Q Zhang Y. Unattended social wants and corporate social responsibility of leading firms: R elationship of intrinsic motivation of volunteering in proposed welfare programs and employee attributes. Corp Soc Responsib Environ Manag. (2018) 25:1029–38. 10.1002/csr.1681

[77] Farooq Q Fu P Liu X Hao Y. Basics of macro to microlevel corporate social responsibility and advancement in triple bottom line theory. Corp Soc Responsib Environ Manag. (2021) 28:969–79. 10.1002/csr.2069

[78] Asante Boadi E He Z Bosompem J Opata CN Boadi EK. Employees' perception of corporate social responsibility (CSR) and its effects on internal outcomes. Service Industries J. (2020) 40:611–32. 10.1080/02642069.2019.1606906

[79] Hur W-M Moon T-W Ko S-H. How employees' perceptions of CSR increase employee creativity: mediating mechanisms of compassion at work and intrinsic motivation. J Busi Ethics. (2018) 153:629–44. 10.1007/s10551-016-3321-5

[80] Kotera Y Taylor E Fido D Williams D Tsuda-McCaie F. Motivation of UK graduate students in education: self-compassion moderates pathway from extrinsic motivation to intrinsic motivation. Curr Psychol. (2021). 10.1007/s12144-021-02301-6. [Epub ahead of print]. 34566390 PMC8455232

[81] David D. How to cultivate gratitude, compassion, pride on your team. Harvard Business Review Digital Articles. (2018). Available online at: https://hbr.org/2018/02/how-to-cultivate-gratitude-compassion-and-pride-on-your-team (accessed February 20, 2018).

[82] Hofmeyer A Taylor R Kennedy K. Fostering compassion and reducing burnout: How can health system leaders respond in the COVID-19 pandemic and beyond? Nurse Educ Today. (2020) 94:104502. 10.1016/j.nedt.2020.104502 32980180 PMC7295512

[83] Simpson AV Farr-Wharton B Reddy P. Cultivating organizational compassion in healthcare. J Health Organ Manag. (2020) 26:340–54. 10.1017/jmo.2019.54

[84] Lown BA Shin A Jones RN. Can organizational leaders sustain compassionate, patient-centered care and mitigate burnout? JHealthcare Manag. (2019) 64:398–412. 10.1097/JHM-D-18-00023 31725567

[85] Perez-Bret E Altisent R Rocafort J. Definition of compassion in healthcare: a systematic literature review. Int J Palliat Nurs. (2016) 22:599–606. 10.12968/ijpn.2016.22.12.599 27992278

[86] Boyatzis RE Smith ML Beveridge AJ. Coaching with compassion: Inspiring health, well-being, and development in organizations. J Appl Behav Sci. (2013) 49:153–78. 10.1177/0021886312462236

[87] De Dreu CK Nijstad BA Van Knippenberg D. Motivated information processing in group judgment and decision making. Pers Soc Psychol Rev. (2008) 12:22–49. 10.1177/1088868307304092 18453471

[88] Eldor L Shoshani A. Caring relationships in school staff: Exploring the link between compassion and teacher work engagement. Teach Teach Educ. (2016) 59:126–36. 10.1016/j.tate.2016.06.001

[89] Mauno S Ruokolainen M Kinnunen U De Bloom J. Emotional labour and work engagement among nurses: examining perceived compassion, leadership and work ethic as stress buffers. J Adv Nurs. (2016) 72:1169–81. 10.1111/jan.12906 26841277

[90] Grant AM. Does intrinsic motivation fuel the prosocial fire? Motivational synergy in predicting persistence, performance, and productivity. J Exp Psychol. (2008) 93:48. 10.1037/0021-9010.93.1.48 18211134

[91] Adnan M Ahmad N Scholz M Khalique M Naveed RT Han H. Impact of substantive staging and communicative staging of sustainable servicescape on behavioral intentions of hotel customers through overall perceived image: A case of boutique hotels. Int J Environ Res Public Health. (2021) 18:9123. 10.3390/ijerph18179123 34501713 PMC8431223

[92] Awan K Ahmad N Naveed RT Scholz M Adnan M Han H. The impact of work–family enrichment on subjective career success through job engagement: a case of banking sector. Sustainability. (2021) 13:8872. 10.3390/su13168872

[93] Chen J Ghardallou W Comite U Ahmad N Ryu HB Ariza-Montes A. Managing hospital employees' burnout through transformational leadership: the role of resilience, role clarity, and intrinsic motivation. Int J Environ Res Public Health. (2022) 19:10941. 10.3390/ijerph191710941 36078657 PMC9518422

[94] Alam T Ullah Z AlDhaen FS AlDhaen E Ahmad N Scholz M. Towards explaining knowledge hiding through relationship conflict, frustration, and irritability: the case of public sector teaching hospitals. Sustainability. (2021) 13:12598. 10.3390/su132212598

[95] Guan X Ahmad N Sial MS Cherian J Han H. CSR and organizational performance: The role of pro-environmental behavior and personal values. Corp Soc Responsib Environ Manag. (2022). 10.1002/csr.2381. [Epub ahead of print].

[96] Peng J Samad S Comite U Ahmad N Han H Ariza-Montes A. Environmentally specific servant leadership and employees' energy-specific pro-environmental behavior: evidence from healthcare sector of a developing economy. Int J Environ Res Public Health. (2022) 19:7641. 10.3390/ijerph19137641 35805297 PMC9266249

[97] Ullah Z Shah NA Khan SS Ahmad N Scholz M. Mapping institutional interventions to mitigate suicides: a study of causes and prevention. Int J Environ Res Public Health. (2021) 18:10880. 10.3390/ijerph182010880 34682627 PMC8535598

[98] Turker D. Measuring corporate social responsibility: a scale development study. J Bus Ethics. (2009) 85:411–27. 10.1007/s10551-008-9780-6

[99] Kristensen TS Borritz M Villadsen E Christensen KB. The copenhagen burnout inventory: a new tool for the assessment of burnout. Work Stress. (2005) 19:192–207. 10.1080/02678370500297720

[100] Schaufeli WB Shimazu A Hakanen J Salanova M De Witte H. An ultra-short measure for work engagement: the UWES-3 validation across five countries. Eur J Psychol Assess. (2019) 35:577. 10.1027/1015-5759/a000430

[101] Tierney P Farmer SM Graen GB. An examination of leadership and employee creativity: the relevance of traits and relationships. Pers Psychol. (1999) 52:591–620. 10.1111/j.1744-6570.1999.tb00173.x

[102] Lilius JM Worline MC Maitlis S Kanov J Dutton JE Frost P. The contours and consequences of compassion at work. J Organ Behav. (2008) 29:193–218. 10.1002/job.508

[103] Han H. Al-Ansi A. Chua B.-L. Ahmad N. Kim J. J. Radic A.. (2022). Reconciling civilizations: eliciting residents' attitude and behaviours for international Muslim tourism and development. Curr Issues Tour. 1–19. 10.1080/13683500.2022.2056003

[104] Ahmad N Ullah Z AlDhaen E Han H Ariza-Montes A Vega-Muñoz A. Fostering advocacy behavior of employees: a corporate social responsibility perspective from the hospitality sector. Front Psychol. (2022) 13. 10.3389/fpsyg.2022.865021 35572254 PMC9093048

[105] Gupta S Nawaz N Tripathi A Muneer S Ahmad N. Using social media as a medium for CSR communication, to induce consumer–brand relationship in the banking sector of a developing economy. Sustainability. (2021) 13:3700. 10.3390/su13073700

[106] Zhang D Mahmood A Ariza-Montes A Vega-Muñoz A Ahmad N Han H. Exploring the impact of corporate social responsibility communication through social media on banking customer e-wom and loyalty in times of crisis. Int J Environ Res Public Health. (2021) 18:4739. 10.3390/ijerph18094739 33946787 PMC8124371

[107] Ullah Z AlDhaen E Naveed RT Ahmad N Scholz M Hamid TA. Towards making an invisible diversity visible: a study of socially structured barriers for purple collar employees in the workplace. Sustainability. (2021) 13:9322. 10.3390/su13169322

[108] Hayes AF. Introduction to mediation, moderation, and conditional process analysis: A regression-based approach. New York: Guilford publications. (2017).

[109] Svergun O Fairlie P. The interrelated roles of corporate social responsibility and stress in predicting job outcomes. J Workplace Behav Health. (2020) 35:193–210. 10.1080/15555240.2020.1775625

